# Rat umbilical cord blood cells attenuate hypoxic–ischemic brain injury in neonatal rats

**DOI:** 10.1038/srep44111

**Published:** 2017-03-10

**Authors:** Keiko Nakanishi, Yoshiaki Sato, Yuka Mizutani, Miharu Ito, Akihiro Hirakawa, Yujiro Higashi

**Affiliations:** 1 Department of Perinatology, Institute for Developmental Research, Aichi Human Service Center, 713-8 Kagiya-cho, Kasugai, Aichi, 480-0392, Japan; 2 Division of Neonatology, Center for Maternal-Neonatal Care, Nagoya University Hospital, 65 Tsurumai-cho, Showa-ku, Nagoya, 466-8560, Japan; 3 Center for Advanced Medicine and Clinical Research, Nagoya University Hospital, 65 Tsurumai-cho, Showa-ku, Nagoya, 466-8560, Japan

## Abstract

Increasing evidence has suggested that human umbilical cord blood cells (hUCBC) have a favorable effect on hypoxic–ischemic (HI) brain injury. However, the efficacy of using hUCBCs to treat this injury has been variable and the underlying mechanism remains elusive. Here, we investigated its effectiveness using stereological analysis in an allogeneic system to examine whether intraperitoneal injection of cells derived from UCBCs of green fluorescent protein (GFP)-transgenic rats could ameliorate brain injury in neonatal rats. Three weeks after the HI event, the estimated residual brain volume was larger and motor function improved more in the cell-injected rats than in the control (PBS-treated) rats. The GFP-positive cells were hardly detectable in the brain (0.0057% of injected cells) 9 days after injection. Although 60% of GFP-positive cells in the brain were Iba1-positive, none of these were positive for NeuroD or DCX. While the number of proliferating cells increased in the hippocampus, that of activated microglia/macrophages decreased and a proportion of M2 microglia/macrophages increased in the ipsilateral hemisphere of cell-injected rats. These results suggest that intraperitoneal injection of cells derived from UCBCs could ameliorate HI injury, possibly through an endogenous response and not by supplying differentiated neurons derived from the injected stem cells.

Despite the recent advances in perinatal care, perinatal hypoxia–ischemia (HI) remains a tragic cause of neonatal death and/or severe neurological disorders^1^. The incidence of perinatal HI remains high even in developed countries (1.3–1.7 per 1000 live births)^2^, and many survivors suffer long-term neurological disabilities. Although brain hypothermia has been a common clinical tool for treating HI brain injury, it is not sufficiently effective in severe cases^3^,^4^. Therefore, the development of novel and efficacious therapies for treating perinatal HI-induced brain injury has long been expected.

Stem cell therapy is a useful treatment for various refractory central nervous system (CNS) diseases^5^. We previously demonstrated that intracerebroventricular injection of neural stem/progenitor cells (NSPCs) together with chondroitinase ABC, which digests glycosaminoglycan chains, significantly decreased the degree of cerebral infarction after perinatal HI injury in a rat model^6^,^7^. However, as a clinical cure, certain difficulties must be overcome in applying intracerebroventricular injection of stem cells derived from human brains.

Among several sources of stem cells, umbilical cord blood cells (UCBCs) provide excellent material, especially for neonates. There are several advantages to using UCBCs as a stem cell source in neonates. First, they are readily available at birth and can be used for autologous transplantation. Second, they can be administered intravenously^8^, and cross the blood-brain barrier^9^. Umbilical cord blood transplantation has been used as a clinical therapy for treating hematological diseases such as leukemia, confirming the establishment of their safety in transplantation. In addition, it is generally accepted that UCBCs are hypo-immunogenic^10^,^11^ and do not induce tumorigenesis *in vivo*^12^.

Several studies using UCBCs for HI-induced brain damage in a neonatal rat/mouse model have been reported^13^,^14^,^15^,^16^,^17^,^18^. Moreover, clinical trials using autologous human UCBCs for neonatal hypoxic-ischemic encephalopathy (HIE) have been initiated^19^,^20^, and its feasibility has been reported^21^. Although many animal studies showed that UCBCs had a favorable effect in treating HI-induced damage, their outcomes appeared variable, ranging from ineffective to efficacious, and the underlying mechanisms remain largely unknown^13^,^14^,^15^,^16^,^17^,^18^. This variation in results might reflect the use of human UCBCs in a rat/mouse model, since human UCBCs could be partly rejected immunologically, resulting in a significant reduction of their migration into the CNS or their effect on the outcome, or various trophic factors derived from the human UCBCs may not have been able to perfectly provoke restoration of the damaged brain because of possible involvement of species-specific factors. For circumventing these issues, it is essential to establish an experimental system of allogeneic transplantation using the same species for donor cells and recipient animals.

Here, for the first step toward investigating the molecular mechanism involved in the effectiveness of using transplanted UCBCs to restore the damaged brain, we first established culture conditions for the expansion of rat UCBCs using several growth factors, and then administered these cells to a rat HI model to examine the effect of the transplantation on the damaged brain tissues both morphologically and functionally. In the present study, we found that intraperitoneal injection of rat UCBCs ameliorated HI-induced brain injury even though the administered UCBCs were hardly detectable in the brain of cell-treated rats. We propose that administered UCBCs attenuate brain injury possibly through an unidentified endogenous response and not by supplying the brain with neurons that had differentiated from the injected stem cells.

## Results

### Expansion of rat mononuclear cells derived from UCBCs

**Figure 1:**
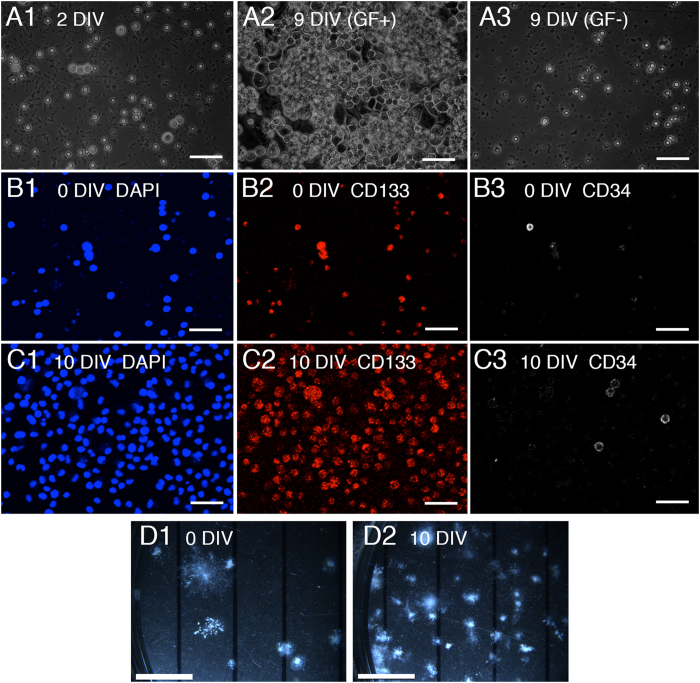
Characterization of mononuclear cells derived from umbilical cord blood cells. (**A**) Representative phase contrast photomicrographs of cultured mononuclear cells (MNCs) from rat umbilical cord blood cells (UCBC) at 2 days *in vitro* (DIV, left), 9 DIV with growth factors (GF+, middle), and 9 DIV without growth factors (GF-, right). Bar, 100 μm. (**B**,**C**) Cultured rat MNC-UCBCs at 0 DIV (**B**) and 10 DIV (**C**) were immunostained using anti-CD133 (middle panels) and anti-CD34 (right panels) antibodies. Nuclei were counterstained with DAPI (left panels). Most proliferated cells at 10 DIV were positive for CD133. Bar, 100 μm. (**D**) Representative photomicrographs of colony-forming unit-granulocyte, macrophage (CFU-GM) assay results using rat MNCs of UCBCs at 0 days (3 × 10^3^ cells/well, left) and expanded cells with growth factors at 10 days *in vitro* (5 × 10^2^ cells/well, right), respectively. Photomicrographs were taken 10 days later (see Methods). Many CFU-GM-positive cells were present in the expanded cells at 10 DIV. Bar, 5 mm.

In order to administer UCBCs from rats harboring a green fluorescent protein (GFP) transgene to HI-injured rats, we first prepared mononuclear cells (MNCs) from UCBCs of a litter of embryonic rats at E19. However, the total number of MNCs obtained was much smaller than the amount needed for the intraperitoneal injection (0.72 ± 0.18 × 10^6^ cells per litter, Table 1) compared to the number of human UCBCs used for rat HI injury models (more than 1 × 10^7^ cells per individual)^13^,^14^,^22^. In order to prepare a larger number of UCBCs, we cultured MNCs from rat UCBCs with growth factors *in vitro*. At 9 days of culture, the total number of cells had increased considerably, while cells cultured without growth factors had not (Fig. 1A). The total cell number increased from 0.72 ± 0.18 to 10.7 ± 1.5 × 10^6^ 10 days after culture *in vitro* (n = 5, Table 1). Most of the proliferating cells were positive for CD133 (a stem cell marker), and some were also CD34-positive (a marker of primitive blood- and bone-marrow-derived progenitor cells)(Fig. 1B,C). The number of progenitor cells estimated by a colony-forming unit-granulocyte, macrophage (CFU-GM) assay increased as well (Fig. 1D). The percentage of colony-forming cells estimated by the CFU-GM assay increased from 0.90 ± 0.12% to 20.2 ± 1.0% (n = 4). Accordingly, the total number of colony-forming cells was estimated to have increased from 0.77 ± 0.21 to 234 ± 40 × 10^4^ (n = 4, Table 1). Hereafter, we refer to the expanded cells as stem-cell-enriched UCBCs (SCE-UCBCs).

### Intraperitoneal injection of SCE-UCBCs reduced the infarct volume after HI

**Figure 2:**
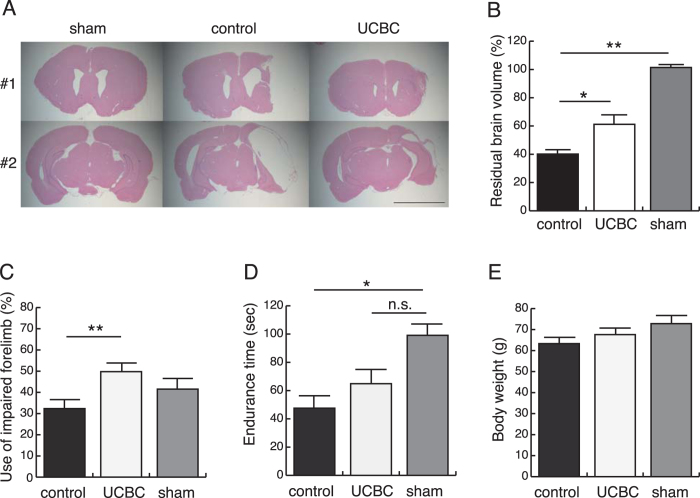
Injection of SCE-UCBCs ameliorated HI-induced brain injury and improved motor function. (**A**) Coronal sections of hypoxic-ischemic rats 21 days after the insult were sliced at Bregma (#1, upper panels) and Bregma-5 mm (#2, lower panels). Typical histological features of the brain can be seen with HE staining of sham (left), control (middle), and SCE-UCBC-treated (right) rats. Bar, 5 mm. (**B**) Estimated residual brain volumes designated as the proportion of the ipsilateral hemisphere relative to the contralateral one. The residual brain volumes of SCE-UCBC-treated rats were significantly larger than those of control rats. (**C**) Cylinder testing was performed 21 days after insult. The frequencies of use of the impaired forelimbs in the SCE-UCBC-treated rats were significantly higher than those of control rats. (**D**) Rotarod performance test. The endurance times of control rats were significantly shorter than those of sham rats, whereas those of SCE-UCBC-treated rats were not significantly different from those of sham rats. (**E**) Body weight at P28. Body weights were comparable among the three groups. Data are expressed as means ± SEM. *p < 0.05, **p < 0.01, n.s.; not significant. (sham-operated, n = 5; control n = 15; UCBC, n = 14).

At postnatal day 7, rats were subjected to HI treatment, and subsequently, SCE-UCBCs from GFP transgenic rats (2 × 10^6^ cells/individual) or vehicle (phosphate-buffered saline, PBS) were intraperitoneally administered to the brain-injured rats 3 days after HI. Three weeks after the insult, the injected rats were subjected to behavioral testing (see below) and histological analyses. Serial coronal sections were prepared and then subjected to HE staining (Supplementary Fig. S1). Representative photomicrographs are shown in Fig. 2A. The infarct areas in SCE-UCBC-injected rats were obviously smaller than those in the control in both Bregma and Bregma-5 mm sections. The residual brain volume was estimated by stereological analysis and designated as the ratio of the ipsilateral (right) hemisphere volume to the contralateral (left) volume (see Methods and Supplementary Information). The absolute value of each hemisphere volume was shown in Supplementary Table S1. The estimated residual brain volumes of the SCE-UCBC-injected rats were significantly larger than those of control rats (control rats, 40.1 ± 2.8%, n = 15 vs. UCBC rats, 61.2 ± 6.9%, n = 14, p < 0.05, Fig. 2B), but were smaller than those of sham rats (UCBC rats, 61.2 ± 6.9%, n = 14, sham rats, 101.4 ± 2.0%, n = 5, not significant, Fig. 2B). When the brain tissues were subdivided into anterior cortex (AC, defined as the cortex from Bregma +5 mm to Bregma), posterior cortex (PC, defined as the cortex from Bregma to Bregma-5 mm), caudate putamen (CPu), and hippocampus (H, Supplementary Fig. S1), the estimated residual volumes in the PC and hippocampus of the SCE-UCBC-injected rats were significantly larger than those of control rats (PC, control rats, 22.5 ± 3.1%, n = 15 vs. UCBC rats, 50.4 ± 9.3%, n = 14, p < 0.05; hippocampus, control rats, 26.2 ± 4.0%, n = 15 vs. UCBC rats, 56.0 ± 8.8%, n = 14, p < 0.05, Supplementary Fig. S1). These results suggested that intraperitoneal injection of rat SCE-UCBCs ameliorates brain injury in the neonatal rat HI model.

### Intraperitoneal injection of SCE-UCBCs ameliorated motor function in HI-injured rats

Many survivors of perinatal HIE suffer long-term neurological disabilities, exhibiting motor function disorders such as cerebral palsy. Therefore, we focused on motor function in the present study. To evaluate the effect of administration of SCE-UCBCs on HI-induced motor deficits, the cylinder test and rotarod performance test were performed using rats at 21 days after insult (P28). The cylinder test is designed to evaluate locomotor asymmetry in rodent models of CNS disorders. The frequency of use of the impaired forelimb during the cylinder test was 32.4 ± 4.7% in HI-injured rats given control PBS (n = 15), while that of HI-injured rats given SCE-UCBCs was comparable with use of the intact forelimb (49.8 ± 2.9%, n = 14, p < 0.01, Fig. 2C). The rotarod performance test is a basic experimental method for evaluating the motor coordination of rodents. The test endurance times of HI-injured rats administered the PBS control were significantly shorter than those of the sham-treated rats (control rats, 47.6 ± 10.1 sec, n = 15, vs. sham rats, 99.1 ± 8.5 sec, n = 5, p < 0.05, Fig. 2D). Those of HI-injured rats administered SCE-UCBCs were not significantly different from those of rats receiving the sham treatment (UCBC rats, 64.9 ± 10.4 sec, n = 14, vs. sham rats, 99.1 ± 8.5 sec, n = 5, not significant, Fig. 2D). Body weights at P28 were comparable among the three groups (control rats, 63.3 ± 1.6 g, n = 15; UCBC rats, 67.6 ± 2.5 g, n = 14; sham rats, 72.8 ± 3.2 g, n = 5, not significant, Fig. 2E). When we analyzed the relationship between the estimated residual brain volume (Fig. 2B) or PC volume (Supplementary Fig. S1) evaluated by stereological analysis using HE staining, and motor function evaluated by the behavioral study (Fig. 2C,D) in all rats of the three groups, the endurance time on the rotarod had some correlation with the residual brain volume and residual PC volume (Supplementary Fig. S2). These results suggest that administration of SCE-UCBCs ameliorates HI-induced motor deficits.

### Intraperitoneally injected SCE-UCBCs slightly migrated into the CNS

**Figure 3:**
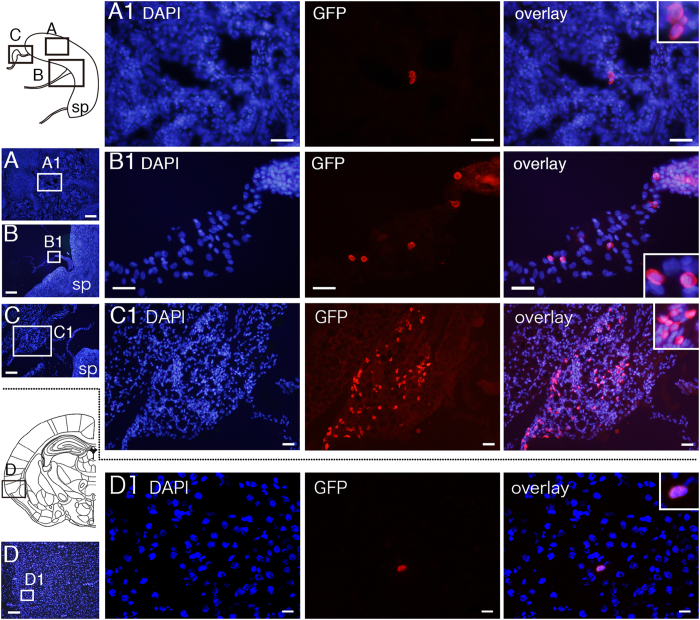
Detection of intraperitoneally injected SCE-UCBCs in the spleen, the tissues around the spleen, and the brain of HI-treated rats. Photomicrographs of the spleen (**A**), the tissues around the spleen (**B**,**C**), and the brain (**D**) of HI-treated rats intraperitoneally injected with GFP-labeled SCE-UCBCs 9 days after injection. Although GFP-positive cells were readily observed in the funicular tissues around the spleen (B1,C1), only a few GFP-positive cells were detectable in the spleen (A1) and the brain (D1) of rats injected with SCE-UCBCs. Boxes A, B, and C in the upper schematic illustration and D in the lower schematic illustration of the coronal section are presented as photographs in **A**,**B**,**C**, and **D**, respectively. Boxes in A–D are magnified as in A1–D1. DAPI (blue), anti-GFP antibody (red) staining, rightmost panel, overlay. Insets show higher magnification views. sp; spleen, Bar, A and C, 200 μm, B, 500 μm, D, 100 μm, A1–C1, 50 μm, and D1, 20 μm.

In order to determine whether the effect of the intraperitoneally injected SCE-UCBCs on the amelioration of HI-induced brain injury was due to the direct supply of functional neurons that had differentiated from the injected stem cells, we examined whether intraperitoneally injected SCE-UCBCs could migrate into the CNS. At postnatal day 7, rats were subjected to HI injury, and subsequently, SCE-UCBCs from GFP transgenic rats (2 × 10^6^ cells/individual) were intraperitoneally administered to the brain-injured rats at 3 days after HI. If intraperitoneally injected SCE-UCBCs enter the bloodstream, they would be expected in the spleen, since the bloodstream flows through it. Four or 9 days after administration of SCE-UCBCs, serial cryosections of the spleen and brain were prepared and subjected to immunostaining using anti-GFP antibodies every 500 μm sections. The GFP-positive cells were barely detected in the spleen (18.8 ± 12.0 and 210 ± 106 cells/individual at 4 and 9 days after administration, respectively, each n = 4, Fig. 3A) but readily observed in the tissue around the spleen (2550 ± 1663 and 2980 ± 1990 cells/individual at 4 and 9 days after administration, respectively, each n = 4, Fig. 3B,C), indicating that the intraperitoneally injected SCE-UCBCs survived in the animals and had migrated into the blood flow. The GFP-positive cells were also detected in the brain (Fig. 3D), but the population was quite low (43.8 ± 15.7 and 114 ± 40 cells/individual, 0.0022% and 0.0057% of injected cells, at 4 and 9 days after administration, respectively, each n = 4). Although they were usually found in brain parenchyma, some were in cerebrospinal fluid. The GFP-positive cells were usually round in shape without protrusions on co-staining with 4′, 6′-diamidino-2-phenylindole (DAPI), an apparently different shape from that of mature neurons.

**Figure 4:**
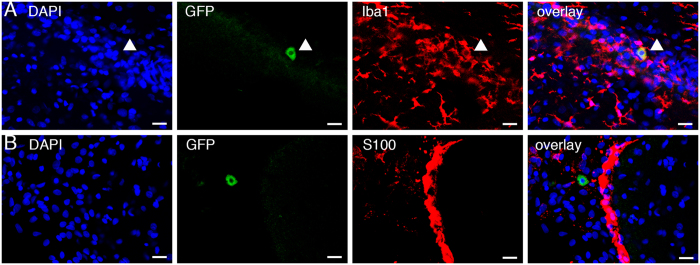
Double staining of intraperitoneally injected SCE-UCBCs in the brain of HI-treated rats. Representative photomicrographs of the double staining of GFP/Iba1 (**A**) and GFP/S100 (**B**) in brains of HI-treated rats intraperitoneally injected with GFP-labeled SCE-UCBCs 7 days after injection. DAPI (blue), anti-GFP antibody (green), and anti-Iba1-(**A**) or S100-(**B**) antibody (red) staining, rightmost panel, overlay. Although some GFP+/Iba1+ cells were detected (**A**, 9 out of 15 GFP+ cells, arrowhead), none of GFP+ cells were positive for S100 (**B**, 0 out of 21 GFP+ cells). Bar, A and B, 20 μm.

Double-staining revealed that some GFP-positive cells in the brain were also positive for Iba1 (9 cells out of 15 GFP-positive cells, 60%, Fig. 4A), suggesting that some of the injected SCE-UCBCs differentiated into microglia/macrophages in the brain. In contrast, GFP-positive cells in the brain were negative for S100 (an astrocyte marker, 0 cells out of 21 GFP-positive cells, 0%, Fig. 4B), NeuroD (an immature neuronal marker, 0 cells out of 10 GFP-positive cells, 0%, see Supplementary Fig. S3), and DCX (another immature neuronal marker, 0 out of 9 GFP-positive cells, 0%). NeuroD- and DCX-positive cells were observed in the dentate gyrus (DG), however, we failed to detect any GFP/NeuroD- or GFP/DCX-double-positive cells in the DG (see Supplementary Fig. S3). These results suggested that amelioration of HI-induced brain injury by SCE-UCBCs is unlikely to be due to a supply of newly differentiated neurons from the injected UCBCs.

### Impact of SCE-UCBC injection on neurogenesis, angiogenesis, and inflammation

**Figure 5:**
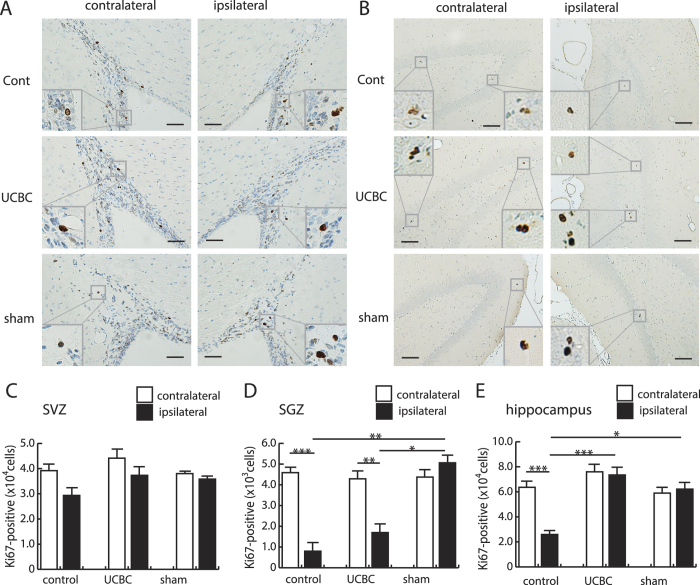
Increase in the number of Ki67-positive proliferating cells in the hippocampus of rats injected with SCE-UCBCs. Representative photomicrographs of anti-Ki67 staining of the subventricular zone (SVZ, **A**) and hippocampus (**B**) of the contralateral (left panels) and ipsilateral (right panels) hemispheres of a control rat (upper panels), a SCE-UCBC-treated rat (middle panels), and a sham rat (lower panels) 3 weeks after insult. Insets show higher magnification views. The rectangles indicate the areas magnified. Bar, A, 50 μm, B, 100 μm. (**C**–**E**) Numbers of Ki67-positive cells in the SVZ (**C**), subgranular zone (SGZ, **D**), and hippocampus (**E**). Data are expressed as means ± SEM. *p < 0.05, **p < 0.01, ***p < 0.001. (sham-operated, n = 5; control n = 15; UCBC, n = 14).

To determine how the injected SCE-UCBCs act to protect damaged neurons, we first carried out an immunohistochemical examination of brains using Ki67 (a proliferation marker), tomato lectin (a marker of blood vessels), and ED1 (an activated microglia/macrophage marker), three weeks after insult, and analyzed the results using stereological methods (see Supplementary Information). Representative photomicrographs of cells stained with anti-Ki67 antibodies are shown in Fig. 5A,B. Numerous Ki67-positive cells were mainly observed in the subventricular zone (SVZ) of lateral ventricles and the subgranular zone (SGZ) of DG, where neurogenesis usually occurs^23^,^24^. The numbers of Ki67-positive cells in the SVZ were not significantly different in either hemisphere among the three groups (Fig. 5C). In addition, there were no significant differences in this number in the ipsilateral SGZ between control and SCE-UCBC-injected rats (Fig. 5D), although the ipsilateral SGZ had significantly fewer Ki67-positive cells than the contralateral SGZ in both groups (control rats, contralateral, 4730 ± 294 vs. ipsilateral, 703 ± 333 cells/individual, n = 15, p < 0.001; UCBC rats, contralateral, 4490 ± 388, vs. ipsilateral, 1840 ± 434 cells/individual, n = 14, p < 0.01, Fig. 5D). In contrast, the number of Ki67-positive cells in ipsilateral hippocampus of SCE-UCBC-injected rats was greater than in control rats (UCBC rats, 73500 ± 6240, n = 14, vs. control rats, 25900 ± 3190 cells/individual, n = 15, p < 0.001, Fig. 5E). These results suggest that the SCE-UCBC injection induced cell proliferation in the ipsilateral hippocampus.

**Figure 6:**
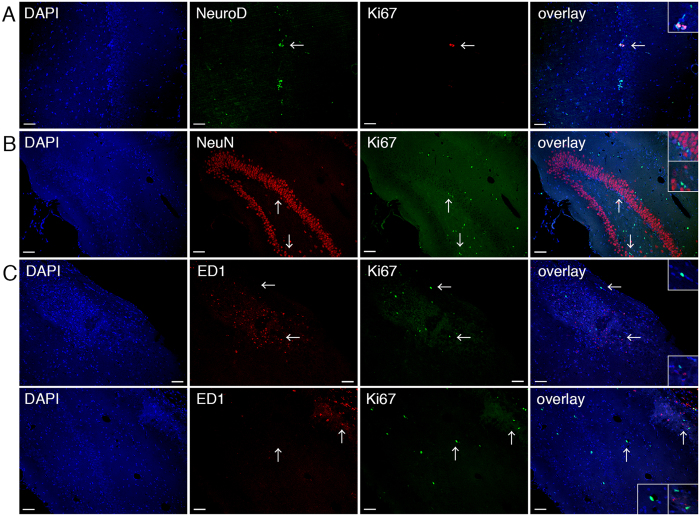
The majority of Ki67-positive cells in the ipsilateral hippocampus were NeuN- and ED1-negative. (**A**) Representative photomicrographs of the double staining of Ki67 and NeuroD in the ipsilateral hippocampus of an SCE-UCBC-treated rat. DAPI (blue), anti-NeuroD antibody (green), and anti-Ki67 antibody (red) staining, rightmost panel, overlay. A small portion of Ki67-positive cells was NeuroD-positive (7 cells out of 132 Ki67-positive cells in the ipsilateral hippocampus, 5.3%, n = 8; 3 cells out of 35 Ki67-positive cells in the ipsilateral SGZ, 8.6%, n = 8). The double-positive NeuroD/Ki67 cells are shown in **A**. (**B**) Representative photomicrographs of the double staining of Ki67 and NeuN in the ipsilateral hippocampus of an SCE-UCBC-treated rat. DAPI (blue), anti-NeuN antibody (red), and anti-Ki67 antibody (green) staining, rightmost panel, overlay. The Ki67-positive cells were NeuN-negative (0 cells out of 114 Ki67-positive cells, n = 3). (**C**) Representative photomicrographs of the double staining of Ki67 and ED1 in the ipsilateral hippocampus of a control rat (upper panels) and SCE-UCBC-treated rat (lower panels). DAPI (blue), anti-ED1 antibody (red), and anti-Ki67 antibody (green) staining, rightmost panel, overlay. The Ki67-positive cells were ED1-negative (0 cells out of 99 Ki67-positive cells in the ipsilateral hippocampus of an SCE-UCBC-treated rat, n = 3). The specimens containing the greatest number of Ki67-positive cells were selectively shown in this figure, making it easier to recognize the type of proliferating cells. Insets show higher magnification views. Arrows indicate the area magnified. Bar, 50 μm.

**Figure 7:**
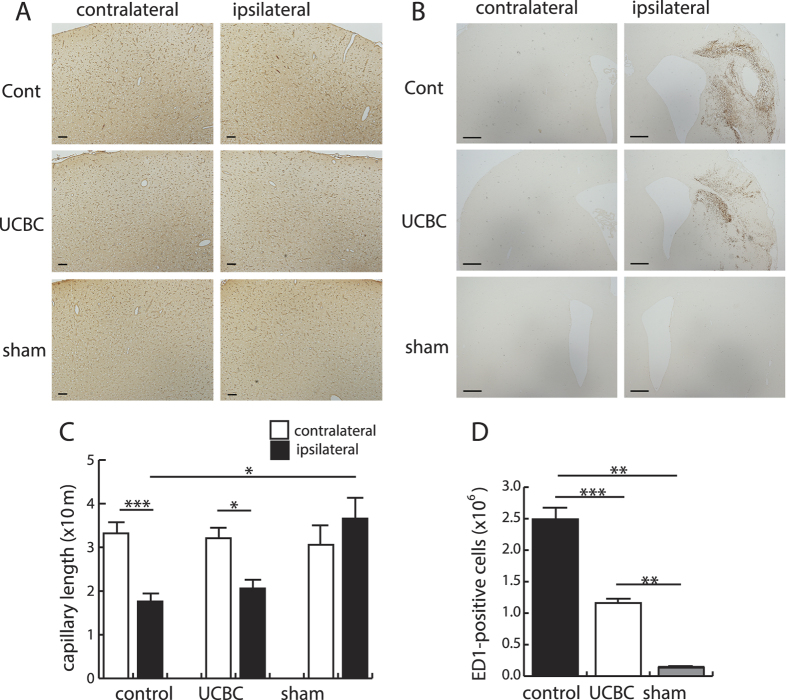
Effect of injection of SCE-UCBCs on vascular formation and the inflammatory response. Representative photomicrographs of the primary/secondary motor cortex stained with tomato lectin (**A**) and the peri-infarct area stained with anti-ED1 antibodies (**B**) in the contralateral (left panels) and ipsilateral (right panels) hemispheres of a control rat (upper panels), SCE-UCBC-treated rat (middle panels), and sham rat (lower panels) 3 weeks after insult. Bar, A, 100 μm, B, 500 μm. (**C**,**D**) The capillary length as estimated using tomato-lectin staining (see Supplementary Information) in the motor cortex (**C**) and the number of ED1-positive cells in the ipsilateral hemisphere (**D**). The number of ED1-positive cells in the ipsilateral hemisphere of SCE-UCBC-treated rats decreased compared to control rats. Data are expressed as means ± SEM. *p < 0.05, **p < 0.01, ***p < 0.001. (sham-operated, n = 5; control n = 15; UCBC, n = 14).

To know the cell type of the induced Ki67-positive cells, we performed the double staining analysis using some cell lineage markers. A small portion of Ki67-positive cells expressed NeuroD in the ipsilateral hippocampus (5.3%, n = 8; 7 cells out of 132 Ki67-positive cells) as well as SGZ of DG (8.6%, n = 8; 3 cells out of 35 Ki67-positive cells, Fig. 6A), the region of adult neurogenesis. However, the majority of Ki67-positive cells detected in the ipsilateral hippocampus of SCE-UCBC-treated rats were positive for neither NeuN (a neuronal marker, 0 cells out of 114 Ki67-positive cells, n = 3, Fig.6B) nor ED1(an activated microglia/macrophage marker, and consult the results of Fig. 7D, 0 cells out of 99 Ki67-positive cells, n = 3, Fig. 6C) 3 weeks after insult. These results indicate that most of the Ki67-positive cells in the ipsilateral hippocampus of SCE-UCBC-treated rats represented in Fig. 5E were not activated microglia/macrophages, though further characterization of those Ki67-positive cells remains to be done.

We then examined the extent of the immunoreactivity to tomato lectin in every 50^th^ paraffin section in order to evaluate vascular formation by injection of the SCE-UCBCs. Using these specimens, we estimated the capillary length via the space balls method (see Supplementary Information) in the primary/secondary motor cortex because the injection of SCE-UCBCs improved motor functions (Fig. 2C,D). Representative photomicrographs of cells stained with tomato lectin are shown in Fig. 7A. Although the total capillary length of the ipsilateral motor cortex was shorter than the contralateral one in both control and SCE-UCBC-injected rats (control rats, contralateral, 33.2 ± 2.5 vs. ipsilateral, 17.7 ± 1.9 m/individual, n = 15, p < 0.001; UCBC rats, contralateral, 32.1 ± 2.4, vs. ipsilateral, 20.6 ± 2.0 m/individual, n = 14, p < 0.05, Fig. 7C), which probably reflects the brain volume lost by infarction, the capillary length of the ipsilateral motor cortex of the SCE-UCBC-injected group was not significantly different from that of the control (Fig. 7C).

Activated microglia/macrophages identified with anti-ED1 antibodies were predominantly detected in the ipsilateral hemisphere, especially around the infarct areas 3 weeks after insult (Fig. 7B). Activated microglia/macrophages in the ipsilateral hemisphere of SCE-UCBC-treated rats were significantly fewer in number than in control rats (UCBC rats, 1.16 ± 0.55 × 10^6^ vs. control rats, 2.49 ± 0.19 × 10^6^ cells/individual, p < 0.001, Fig. 7D). It should be noted that, at one week after insult, ED1-positive cells were detected in both hemispheres of control and SCE-UCBC-treated rats (Supplementary Fig. S4). Numbers of ED1-positive cells in SCE-UCBC-treated rats one week after insult were also lower than in control rats in both hemispheres (contralateral, UCBC rats, 21.4 ± 1.5 vs. control rats, 43.8 ± 2.4 cells/mm^2^; ipsilateral, UCBC rats, 103.2 ± 49.4 vs. control rats, 198.0 ± 27.2 cells/mm^2^; n = 2, Supplementary Fig. S4). Double staining with anti-Ki67 and anti-ED1 antibodies showed that the majority of Ki67-positive proliferating cells induced in the ipsilateral hippocampus of SCE-UCBC-treated rats were not these activated microglia/macrophages as shown already in Fig. 6C.

Recently, it has become widely accepted that not only macrophages but also microglia can be divided into two distinct types, depending on their pro-inflammatory (M1) and anti-inflammatory (M2) status^25^,^26^. To investigate the impact of SCE-UCBC injection on the microglial M1 or M2 status, double-staining of anti-Mannose receptors (M2 marker) and anti-Iba1 (pan-microglia marker) was performed. As shown in Supplementary Fig. S5, the ratio of mannose receptor-positive to Iba1-positive cells in the area of the ipsilateral lesion increased in SCE-UCBC-treated rats 9 days after administration (control, 8.4 ± 1.1%, n = 3, vs. UCBC rats, 33.1 ± 12.1%, n = 4, p < 0.05, Supplementary Fig. S5). These results suggested that an endogenous inflammatory response appears to be involved in the neuroprotective effects of SCE-UCBC injection. Taken together, the injection of SCE-UCBCs increased proliferating cells in the ipsilateral hippocampus, prevented accumulation of activated microglia/macrophages, promoted accumulation of M2-type microglia/macrophages at the injured sites, and ameliorated HI-induced brain injury.

## Discussion

The therapeutic efficacy of using UCBCs to treat HI-induced brain injury has appeared to vary depending on the experimental conditions employed. In the present study, however, by using allogeneic transplantation where immunogenicity should be much lower than xenotransplantation and the species-specific effects could be avoided, we successfully demonstrated that the administered UCBCs attenuated brain injury to a degree morphologically and functionally comparable to that achieved with the previous xenotransplantation model^13^,^17^.

In a previous report using xenogeneic transplantation, human MNC-UCBCs were used at a dose of 1 × 10^7^ cells/individual in a neonatal HI rat model^13^,^14^,^22^. In the present study, however, we injected expanded UCBCs at a dose of 2 × 10^6^ cells/individual because the expanded UCBCs contained more colony-forming cells (20% of the total cell number) than did the human MNC-UCBCs (less than 0.4% of the total cell number)^27^. Consistent with this, the efficacy of UCBCs in the amelioration of neuronal function in HI rats was reported to be dose-dependent^17^. Repeated administration of mesenchymal stem cells (MSC) to HI rats appeared to have a synergetic effect with high-dose administration, leading to greater repair of the brain damage^28^. Therefore, the preparation of expanded colony-forming cells might allow us to administer more stem cells or to carry out repeated administration, allowing for greater protection of damaged neurons in HI patients.

In the present study, we chose IP injection of SCE-UCBCs as the route of administration because it was the surest way to administer these cells into the HI rats. Considering their clinical application, several routes can be used to administer stem cells such as intravenous (IV), intraperitoneal (IP), and intranasal delivery. IP injection is the most common route for systematic administration in animals and is technically simple, although the rate of absorption by IP is usually only 25–50% as rapid as that via the IV route^29^. Peritoneum has been utilized to perform peritoneal dialysis in patients with renal failure, and clinical research related to IP administration of anticancer drugs is currently underway^30^. Actually, IP and IV deliveries are not comparable in terms of drug efficacy and cell distribution in neonatal HI mice^31^. Some hematopoietic progenitor cells injected intravenously have been reported to become trapped in the pulmonary circulation^32^. Intranasal delivery of umbilical cord-derived MSCs has been reported to preserve myelination in perinatal brain damage^33^, but some device to prevent sneezing might be required in order to administer stem cells reliably. It is possible that, IP delivery might increase unfavorable loss of UCBCs that migrate to organs other than the brain. However, in the present study, despite detecting only a few transplanted cells in the brain, we were able to achieve a reduced infarct volume and improved motor function in HI rats intraperitoneally injected with these cells, indicating that a massive intrusion of administered UCBCs into the brain might not always be necessary for functional improvements following HI injury. The optimal route of stem cell transplantation for neonatal brain injury should be determined in future.

We administered the SCE-UCBCs to HI rats at the subacute phase (3 days after insult) and examined the efficacy on amelioration of the brain injury. We previously showed that administration of human UCBCs at the acute phase (6 hrs) reduced apoptosis and oxidative stress in a neonatal rat HI model, but this effect was only transient^22^. Administration of human MNCs from bone marrow in the acute phase also had a lesser effect in an adult stroke model^34^. Administering mesenchymal stem cells and neural stem cells at the subacute phase (3 days) after brain damage was also reported to be beneficial in terms of the functional outcome and cell engraftment^28^,^35^. Thus, administration of stem cells in the subacute phase might be more beneficial and have a sustained effect on the functional outcome following HI injury.

In the present study, most of the injected UCBCs did not enter the brain, even under the low immunogenicity (Fig. 3D). Some studies have shown that many human UCB-derived cells are able to migrate into the brain and around the lesion area^13^,^17^. However, using a middle cerebral artery occlusion (MCAO) stroke model, Borlongan *et al*. were not able to detect human UCBCs in the brains of adult rats that were intravenously transplanted with these cells, in spite of a reduction in the cerebral infarct size^36^. We performed a quantitative analysis and found only a small portion of GFP-positive cells in the brain (43.8 ± 15.8 and 114 ± 40 cells/individual, 0.0022% and 0.0057% of the injected cells, respectively) compared to those of the tissues around the spleen (2550 ± 1663 and 2980 ± 1990 cells/individual, 0.12% and 0.15% of the injected cells, respectively) at 4 and 9 days after administration, respectively, indicating that the engraftment of these administered cells was more dominant in tissues other than the brain (Fig. 3). In addition, 60% of the GFP-positive cells in the brain were Iba1-positive (Fig. 4A) and none were NeuroD or DCX-positive (Supplementary Fig. S3B,C), indicating that injected UCBCs are unlikely to differentiate into neurons. Recently, paracrine mechanisms in adult stem cell therapy following acute myocardial infarction have been postulated, that is, the transplanted stem cells release soluble factors that engage in paracrine-like activity, and contribute to cardiac repair and regeneration^37^. Administration of exosomes, which are nanometer-sized membranous vesicles released into surrounding bio-fluids, derived from human umbilical cord MSCs improved cardiac systolic function in an acute myocardial infarction model^38^. Likewise, it is possible that the UCBCs engrafted in other tissues or their differentiated progeny likely release a certain trophic factor(s) and give rise to an unidentified endogenous response to reduce brain injury. This is supported by observations that the significant increase in Ki67-positive cells in the ipsilateral hippocampus and decreased mobilization of ED1-positive cells in the lesion area were specifically detected in the SCE-UCBC-treated rats (Figs 5E and 7D).

The effectiveness of the administered UCBCs was considered to be possibly due to a release of neurotrophic factors that promote endogenous neurogenesis and angiogenesis, prevent loss of neuronal cells, and regulate inflammatory response^16^,^18^,^34^,^39^,^40^,^41^,^42^. In the present study, we failed to detect any significant differences between the control and SCE-UCBCs-injected rats in the capillary length of the ipsilateral motor cortex (Fig. 7C) and in numbers of Ki67-positive proliferating cells in the SVZ and SGZ (Fig. 5C,E). However, we cannot eliminate the possibility that UCBCs induce neovascularization and/or neurogenesis in the early phase after administration since we performed these studies 3 weeks after insult, which might be too late to examine the neovascularization and neurogenesis precisely. Further study will be required to clarify whether administration of UCBCs enhances endogenous neurogenesis and neovascularization at much earlier phase in neonatal HI injury.

In the SCE-UCBCs-injected rats, mobilization of ED1-positive cells decreased in the area of the ipsilateral lesion (Fig. 7D). The ED1 antigen is expressed by the majority of tissue macrophages and also used as a marker of activated microglia. Infiltration of myeloid cells from the periphery into the ischemic brain has been performed using bone marrow chimeric mice expressing EGFP and immune cell subset markers^43^,^44^, and the permeability of the blood-brain barrier was reported to increase in certain pathological conditions such as neonatal hypoxic ischemic encephalopathy (HIE)^45^. Given that there are no reliable microglia-specific markers at present, it is difficult to discriminate between microglia and peripheral monocytes/macrophages^46^. Therefore, it is possible that the ED1-positive cells in the present study included not only activated microglia but also infiltrated myeloid cells such as macrophages that originated in the periphery. Despite these facts, it is interesting to note that administration of UCBCs increased the proportion of M2-type (anti-inflammatory) microglia/macrophages in the lesion area (Supplementary Fig. S5C). M1 (pro-inflammatory)-polarized microglia express pro-inflammatory cytokines and mediators such as IL-1β and TNF-β, whereas M2-polarized microglia express anti-inflammatory cytokines and their receptors^25^,^26^. Given that some GFP-positive cells detected in the brain were Iba1-positive (Fig. 4A), secretory factors from the injected stem cells or their differentiated progeny might regulate the balance in the numbers of M1 and M2 microglia/macrophages in the lesion area and might contribute at least in part to improving brain function after an HI insult.

In summary, a single intraperitoneal injection of rat stem-cell-enriched UCBCs at 3 days after insult attenuated morphological or functional HI brain injury. Since the number of GFP-positive UCBCs in the brain was quite low, it is unlikely that UCBCs attenuate brain injury by supplying the brain with neurons that had differentiated from the injected stem cells. We propose that UCBCs engrafted in tissues other than the brain may attenuate brain injury, possibly through an endogenous response. Identification of the trophic effects of UCBCs could provide the information needed to develop a promising therapy for HI injury.

## Methods

### Hypoxic-ischemic brain injury (HI)

All experimental animal protocols in the present study were approved by the Review Board of the Institute for Developmental Research, Aichi Human Service Center, and were carried out according to the guidelines for animal research of the Neuroscience Society of Japan to minimize the number of animals used as well as their suffering. Hypoxic-ischemic brain injury (HI) was produced in Sprague-Dawley (SD) rats (Japan SLC, Inc.) at postnatal day 7 (P7), according to the method of Rice *et al*.^47^ as described previously^6^. After the ligation of the right carotid artery under isoflurane inhalation, the pups were allowed to rest for 2 hrs (1 hr in a chamber at 33 °C and 1 hr with their dam), and were then exposed to a hypoxic environment of 8% O_2_ at 33 °C for 2 hrs. All the pups in a litter underwent ligation or a sham operation. The mortality rate within 3 days of the insult was 17.4%. Three days later, the pups within each litter were divided into three groups (control, cell-treated, and sham), and injected intraperitoneally with rat SCE-UCBCs (2 × 10^6^ cells/0.2 mL, n = 14, see below) or PBS (0.2 mL, n = 15) as a control. The pups were usually alternately allocated to a control group or cell-treated group following the order of the operation. The control rats in all litters had a similar infarct volume, indicating that the effect of the litter as a factor was negligible. None of the pups died after injection with the cells or PBS. The sham rats (n = 5) underwent neither right carotid artery ligation nor hypoxia, and were injected with PBS. Since the sham group was included as a reference, the number of samples in this group was as small as 5.

### Isolation and culture of rat mononuclear cells derived from UCBCs

Mononuclear cells were obtained from UCBCs of rat fetuses harboring a GFP transgene (SD-Tg(CAG-EGFP)CZ-004Osb, Japan SLC, Inc.) according to the method of Migishima *et al*.^48^. The expression of GFP in these rats is driven by the chicken-β-actin promoter and cytomegalovirus enhancer (CAG promoter)^49^ and the neural stem/progenitor cells from these rats were confirmed to be GFP-positive in a previous study^6^. Mononuclear cells were isolated using Ficoll-Conray solution (Lymphosepar I, Immuno-Biological Laboratories) or Ficoll-Paque PLUS solution (GE Healthcare) and were maintained in Stemline hematopoietic stem cell expansion medium (Stemline I, 0189, Sigma-Aldrich Co.) containing 4 mM glutamine (Gibco, Life Technologies), 50 U/ml penicillin G, 0.025 mg/ml streptomycin (Meiji Seika Ltd.), under 95% ambient air, 5% CO_2,_ and an H_2_O-saturated atmosphere at 37 °C. Mononuclear cells were expanded with a cocktail of growth factors consisting of 20 ng/ml each of rat stem cell factor (SCF), rat thrombopoietin (TPO), human Flt3-ligand, and human IL-6 (all purchased from PeproTech Inc.). Eight to ten days after cultivation, cells were collected by pipetting in PBS and cryopreserved with Cellbanker-3 (Nippon Zenyaku Kogyo Co., Ltd.) until use. Immediately prior to administration, cells were thawed at 37 °C, washed once in PBS, and resuspended in PBS at a density of 1 × 10^7^ cells/ml. The viability of frozen cells of each preparation was estimated by comparing the cell number after thawing to that before freezing, and was 62.5 ± 4.9% (n = 5).

### Immunocytochemical procedures

Immunocytochemical procedures were performed as described previously^50^. Briefly, cells were collected by pipetting in PBS, plated on coverslips coated with poly-L-lysine (Sigma-Aldrich) and fixed. Cells were concurrently incubated with primary antibodies (rabbit anti-CD133, Abcam; goat anti-rat CD34, R&D Systems) at 4 °C overnight. The nuclei of cells were counterstained with DAPI (Sigma).

### CFU-GM assay

In order to determine the number of rat colony-forming-unit-granulocyte, macrophage (CFU-GM) progenitors, MethoCult^®^ (GF R3774, StemCell Technologies, USA) was used according to the manufacturer’s instructions. Briefly, 0.3 ml of cell suspension (3 × 10^4^ cells/ml for 0 DIV, n = 4, or 5 × 10^3^ cells/ml for 10 DIV, n = 4) was added to 3 ml of MethoCult^®^. Using a 2.5 ml syringe, 1.1 ml of the MethoCult^®^ mixtures was dispensed to each of two wells of a 6-well plate and cultured. Ten days later, the total number of colony-forming cells (each colony containing more than 30 cells) in each well was counted. At least 4 independent experiments were carried out and their averages are shown in Table 1.

### Behavioral tests

#### Cylinder test

To evaluate locomotor asymmetry, a cylinder test was performed 21 days after the insult^51^. Briefly, animals (control rats, n = 15, SCE-UCBC-treated rats, n = 14, sham rats, n = 5) were individually placed in an open-top clear glass cylinder (18 cm in diameter and 25 cm in height) and their forelimb activity while rearing against the cylinder wall was recorded for 5 min. The use of the right or left forelimb was defined as the placement of the whole palm on the wall. The frequency of use of the impaired forelimb was determined as (the number of impaired forelimb contacts)/(the number of impaired + non-impaired forelimb contacts), and expressed as a percentage.

#### Rotarod performance test

To evaluate motor coordination, a rotarod performance test was performed using a Rota-rod Treadmill for Rat and Mouse (MK-630B, Muromachi Kikai Co., Ltd., Tokyo, Japan) according to the manufacturer’s instructions. Following the cylinder test, animals (control rats, n = 15, SCE-UCBC-treated rats, n = 14, sham rats, n = 5) were placed on the rod and their forced motor activity examined. The rotation of the rod was accelerated gradually from 4 rpm to 40 rpm for 5 min. The endurance time, that is, the duration of running until the animals fell from the rotating rod, was measured. When an animal continued to run for more than 3 min, the session was considered complete and was terminated. Three sessions were performed with a 30-min break between each session. The endurance time of each animal was determined as the average of three sessions.

### Histological and immunohistochemical procedures

Histological and immunohistochemical procedures were performed as described previously^6^. After the behavioral tests, rats (control, n = 15, SCE-UCBC-injected rats, n = 14, sham, n = 5) were deeply anesthetized using isoflurane inhalation and intracardially perfusion-fixed with 4% paraformaldehyde in 0.1 M phosphate buffer. Brains were dehydrated, embedded in paraffin, and cut into 5-μm-thick serial coronal sections. After deparaffinization, hematoxylin-eosin (HE) staining was performed. For immunohistochemical experiments, antigen retrieval was performed by heating the sections at more than 90 °C for 10 min in 10 mM citrate buffer (pH 6.0). After blocking, sections were incubated overnight at 4 °C with primary antibodies (rabbit anti-Ki67, Abcam; a mouse anti-rat CD68, ED1, EMD Millipore). The sections were subsequently incubated with the appropriate biotinylated secondary antibodies (Vector Laboratories). For lectin staining, sections were incubated with biotinylated *Lycopersicon esculentum* (tomato) lectin (Vector Laboratories) in PBS. After endogenous peroxidase activity was inhibited by treatment with 3% H_2_O_2_ in TBS for 10 min, the sections were then treated with avidin–biotin–peroxidase complex (Vectastain ABC Elite kit, Vector Laboratories), followed by peroxidase detection for 15 min (0.01% 3,3′-diaminobenzidine and 0.01% H_2_O_2_). For double-staining, the primary antibodies (mouse anti-NeuN, Millipore; goat anti-NeuroD, Santa Cruz Biotechnology) were incubated concurrently and appropriate secondary antibodies were used.

Since we could not obtain reliable results in detecting GFP-positive cells with paraffin-embedded brain sections at 3 weeks after insult, we conducted another experiment using frozen sections. For quantification of GFP-positive cells in the tissue, the HI rats injected with SCE-UCBCs (2 × 10^6^ cells/individual, n = 8) or PBS (n = 7) 3 days after insult were deeply anesthetized using isoflurane inhalation and intracardially perfusion-fixed with 4% paraformaldehyde 4 days or 9 days after injection. After post-fixation for 15 hrs, the brains and spleens were cryopreserved at −80 °C, and cut into 20-μm-thick serial coronal sections. After blocking the nonspecific binding, the sections were incubated overnight at 4 °C with mouse monoclonal anti-GFP (Abcam) or rabbit polyclonal anti-GFP (MBL) antibodies, and subsequently incubated with the appropriate secondary antibodies containing 1% donkey serum and 10% rat serum. The specificity of these antibodies for GFP was confirmed by the negative staining of primary antibodies in a brain section from a PBS-injected control rat (Supplementary Fig. S6). These antibodies detected a comparable number of signals in the brain, eliminating the possibility that the signals were non-specific. Erratic GFP-immunopositive signals were sometimes observed in the penumbra of the infarct area, even in PBS-injected control rats, therefore, we did not include these cells as positive.

We detected quite a few GFP-positive cells in the brain (114 ± 40 cells/individual, usually 0–1 cells/10 sections), therefore, for double staining with anti-GFP antibodies, the HI rats were injected with a 5 times larger number of SCE-UCBCs (1 × 10^7^ cells/individual, n = 2) 3 days after the insult, brains were perfusion-fixed at 7 days after administration, and cryosections were prepared. Goat polyclonal anti-Iba1 (Abcam), anti-NeuroD (Santa Cruz), rabbit polyclonal anti-S100 (Dako), and anti-DCX antibodies (Abcam) were concurrently incubated with anti-GFP antibodies (MBL or Abcam). For double staining with anti-NeuroD or anti-DCX, antigen retrieval was performed.

For double staining with anti-mannose receptor and anti-Iba1, cryosections obtained 9 days after administration were immunostained with mouse monoclonal anti-mannose receptor (Abcam) and goat polyclonal anti-iba1 (Abcam) antibodies.

### Quantification and stereological analysis of immunohistochemical staining

Quantification and stereological analysis of immunohistochemical staining is described in Supplementary Information.

### Statistical Analysis

All data are presented as means ± standard error (SEM). All data and sections were examined and photographed by one of the investigators, and were analyzed by two individuals who worked independently and blindly as to their classification. The statistical differences in each outcome among the three groups (Figs 2 and 7D, and Supplementary Fig. S1) and the six groups (Figs 5 and 7C) were compared using the Kruskal-Wallis test followed by the Steel-Dwass test. For Supplementary Fig. S5C,D, the unpaired Welch t-test was used. A two-sided P < 0.05 was considered statistically significant. All the analyses were performed using SAS software (version 9.3; SAS Institute Inc., Cary, NC, USA).

## Additional Information

**How to cite this article:** Nakanishi, K. *et al*. Rat umbilical cord blood cells attenuate hypoxic–ischemic brain injury in neonatal rats. *Sci. Rep.* **7**, 44111; doi: 10.1038/srep44111 (2017).

**Publisher's note:** Springer Nature remains neutral with regard to jurisdictional claims in published maps and institutional affiliations.

## Supplementary Material

Supplementary Information

## Figures and Tables

**Table 1:** Characterization of expanded mononuclear cells from rat umbilical cord blood.

	0 DIV	10 DIV	proliferation (times)
Total cell number(per litter) (x 10^6^ cells)	0.72 ± 0.18^a^(n = 6)	10.7 ± 1.5^a^(n = 5)	16.4 ± 2.4
CFU-GM assay (%)(% of colony-forming cells)	0.90 ± 0.12^b^(n = 5)	20.2 ± 1.04^b^(n = 4)	22.7 ± 4.9
Total colony-forming cells(per litter) (x 10^4^ cells)	0.77 ± 0.21(n = 5)	234 ± 40.4(n = 4)	433 ± 87.3
CD133+/DAPI (%)	54.7 ± 10.8^c^(n = 4)	77.0 ± 8.1^c^(n = 4)	
CD34+/DAPI (%)	16.4 ± 4.5^d^(n = 4)	3.8 ± 0.3^d^(n = 4)	

Data are expressed as means ± SEM. ^a,b^p < 0.001, ^c^Not significant, ^d^p < 0.05.
