# Supplementary material for: Rat umbilical cord blood cells attenuate hypoxic–ischemic brain injury in neonatal rats
Source: Sci Rep. 2017 Mar 10;7:44111. doi: 10.1038/srep44111 (PMC5345001; doi:10.1038/srep44111)
Supplement: Supplementary Information [file srep44111-s1.pdf]

Supplementary Information

Rat umbilical cord blood cells attenuate hypoxic–ischemic brain injury in neonatal rats.

By Keiko Nakanishi, Yoshiaki Sato, Yuka Mizutani, Miharu Ito, Akihiro Hirakawa, Yujiro Higashi

Quantification of immunocytochemical staining

Counting of immunopositive cells was performed using the public domain Image J program (Rasband, 1997; Schneider et al., 2012) after taking photomicrographs. The photomicrographs in each experiment were taken under the same conditions and the images were changed into gray scale with Photoshop software (Adobe Systems Inc., San Jose, CA, USA), and analyzed at an equal threshold in Image J.

For detection of CD133-and CD34-positive cells, fluorescent images of at least 5 fields per coverslip (x20 objective) were obtained using a confocal laser scanning microscope (Fluoview FV1000, Olympus, Japan), and the acquired images were subjected to particle analyses using Image J software. The number of positive cells in each field was counted, averaged, and the number expressed as the percentage of the total number of DAPI-positive cells. Four independent experiments were carried out and their averages are shown in Table 1.

Stereological measurement of histological staining

In order to estimate the residual brain volume, every 100th paraffin-embedded section from the whole brain were stained with Hematoxylin-eosin (HE, typically 12 sections / animal, Supplementary Figure S1A), then serial morphological images were acquired using a digital camera (DFC310FX, Leica microsystems, Wetzlar, Germany) under low magnification (x 7). The border of each hemisphere was outlined and the area was measured using the public domain Image J program. The volumes of each hemisphere were calculated from the HE-stained areas according to the Cavalieri's principle using the following formula: $V = \Sigma A \times P \times T$, where V = the total volume, ΣA = the sum of area measurements, P = the inverse of the sampling fraction, and T = the section thickness, as described previously[22]. The absolute value of each hemisphere was shown in Supplementary Table S1. The estimated residual brain volume was designated as the ratio of the ipsilateral (right) hemisphere volume to

contralateral (left) volume.

*Quantitative detection of GFP-positive cell*s

For estimating the migration of GFP-positive cells into the brain and spleen, every 25th cryosection (typically 20 sections in the brain and 10 sections in the spleen) was stained with anti-GFP antibodies and counted the number of GFP-positive cells in each section using the Fluorescent microscopy (Eclipse E800, Nikon Instruments, Japan). The total number of GFP-positive cells were calculated according to the Cavalieri's principle using the following formula: $tN = \Sigma n \times P$, where tN = the total number, Σn = the sum of the number of positive cells in each section, and P = the inverse of the sampling fraction.

Stereological quantification of immunohistochemical staining

In order to stereological quantification of Ki67-positive cells in the SVZ and hippocampus, every 50th paraffin sections (approximately from 1.7 mm anterior to 0.8 mm posterior to the Bregma for SVZ, and from 2.8 mm to 5.8 mm posterior to the Bregma for hippocampus, Paxinos and Watson, 1996) were immunostained with anti-Ki67 antibodies. The SVZ and SGZ were outlined under low magnification (x100, Osato et al., 2010), then the positive cells were counted in each area under high magnification (x400) using StereoInvestigator version 11 stereology software (MicroBrightField Europe EK, Magdeburg, Germany) as described[22]. The total number of Ki67-positive cells was calculated according to the Cavalieri's principle. The number of Ki67-positive cells in the hippocampus, which includes DG, hippocampus proper (CA1, CA2, and CA3), subiculum, presubiculm, parasubiculum, and fimbria, was calculated using the design-based stereological analysis of a region of interest in StereoInvestigator version 11 stereology software according to the manufacturer's instruction.

For estimating the capillary length, the space balls method (Calhoun and Mouton, 2001; Lee et al. 2005) was performed using StereoInvestigator version 11 stereology software. Every 50th paraffin section containing the primary/secondary motor cortex (approximately from 4.2 mm to 0.2 mm anterior to the Bregma, Paxinos and Watson, 1996) was stained with tomato lectin and analyzed. After the primary/secondary motor cortex was outlined, virtual hemispheres were placed within

the sections at all microscopic sampling sites, and the intersections between the hemispheres and the lectin-positive tubular objects under study were counted according to the manufacturer's instruction. Total capillary length in ipsilateral or contralateral motor cortex was calculated by the software and compared.

For counting the number of ED1-positive cells, only the ipsilateral hemisphere was investigated because the ED1-positive cells were found exclusively around the injury in the ipsilateral hemisphere 3 weeks after insult (Fig. 7B). Every 100th paraffin section (approximately from 4.2 mm anterior to 5.8 mm posterior to Bregma, Paxinos and Watson, 1996) was stained with anti-ED1 antibody and the total number of ED1-positive cells in a ipsilateral hemisphere was calculated by the design-based stereological analysis of a region of interest in StereoInvestigator version 11 stereology software.

Quantification of immunohistochemical staining

For counting the ED1-positive cells one week after insult (Supplementary Fig. S4B), the number of ED1-positive cells in the section at Bregma and Bregma-5mm was counted using particle analysis in Image J as described and represented as density.

To quantify the number of mannose receptor- and Iba1-positive cells, fluorescent images of them in peri-infarct area (at least 3 fields per section, every 25th cryosection, typically more than 8 sections per individual) were obtained (x20 objective) using a confocal laser scanning microscope, and the acquired images were subjected to particle analysis using Image J software as described. The number of mannose receptor- or Iba1-positive cells in each field was counted and the number expressed as percentage of the total number of Iba1- or DAPI-positive cells, respectively.

Reference

Rasband, W.S. *ImageJ, U. S. National Institutes of Health, Bethesda, Maryland, USA* (1997-2012). Available at: http://imagej.nih.gov/ij/

Schneider, C.A., Rasband, W.S. & Eliceiri, K.W. NIH Image to ImageJ: 25 years of image analysis. *Nature Methods* **9**, 671-675 (2012).

Paxinos, G. & Watson, C. *The Rat Brain in Stereotaxic Coordinates, 3rd edn.* (Academic Press, 1996).

Osato, K. et al. Apoptosis-inducing factor deficiency decreases the proliferation rate and protects the subventricular zone against ionizing radiation. *Cell Death Dis.* **1**:e84 (2010).

Calhoun, M.E. and Mouton, P.R. Length measurement: new developments in neurostereology and 3D imagery. J Chem Neuroanat 21, 257-265, 2001.

Lee, G.D. et al. Stereological analysis of microvascular parameters in a double transgenic model of Alzheimer's disease. Brain Res Bulletin 65, 317-322, 2005.

Supplementary Table S1   Absolute values of residual brain volume

	Control (n = 15)	UCBC (n = 14)	Sham (n = 5)
Total brain volume			
Contralateral (mm^3)	171.3 ± 5.6	172.1 ± 5.5	163.9 ± 8.5
Ipsilateral (mm^3)	69.2 ± 5.8	103.8 ± 10.9	166.2 ± 9.2
Ipsi/Contra (%)	40.1 ± 2.8 [a,f]	61.2 ± 6.9 [a]	101.4 ± 2.0 [f]
Anterior cortex (AC) volume			
Contralateral (mm^3)	26.3 ± 3.7	25.7 ± 4.2	26.8 ± 5.5
Ipsilateral (mm^3)	10.8 ± 1.1	14.9 ± 3.3	26.2 ± 4.8
Ipsi/Contra (%)	47.5 ± 4.9 [g]	60.5 ± 7.9	99.7 ± 3.7 [g]
Posterior cortex (PC) volume			
Contralateral (mm^3)	43.6 ± 4.0	46.0 ± 4.3	44.9 ± 7.1
Ipsilateral (mm^3)	11.0 ± 2.0	21.7 ± 3.6	43.7 ± 7.4
Ipsi/Contra (%)	22.5 ± 3.1 [b, h]	50.4 ± 9.3 [b]	96.8 ± 2.1 [h]
Total cortex volume			
Contralateral (mm^3)	69.9 ± 2.2	71.7 ± 2.1	71.7 ± 2.8
Ipsilateral (mm^3)	21.8 ± 2.2	36.6 ± 5.2	69.9 ± 3.7
Ipsi/Contra (%)	30.7 ± 2.7 [i]	52.4 ± 8.4	97.3 ± 2.2 [i]
Caudate putamen (CPu) volume			
Contralateral (mm^3)	5.8 ± 1.0	5.9 ± 0.9	7.1 ± 0.7
Ipsilateral (mm^3)	2.2 ± 0.5	2.8 ± 0.6	7.2 ± 0.8
Ipsi/Contra (%)	34.7 ± 4.3 [j]	51.4 ±10.3 [c]	102.7 ± 11.2 [c, j]
Hippocampus volume			
Contralateral (mm^3)	14.4 ± 1.1	13.8 ± 1.2	12.7 ± 2.3
Ipsilateral (mm^3)	3.7 ± 0.7	7.4 ± 1.3	13.3 ± 1.7
Ipsi/Contra (%)	26.2 ± 4.0 [d, k]	56.0 ± 8.8 [d, e,]	109.0 ± 8.7 [e, k]

[a - e] $p < 0.05$, [f - k] $p < 0.01$.   Data are expressed as means ± SEM.

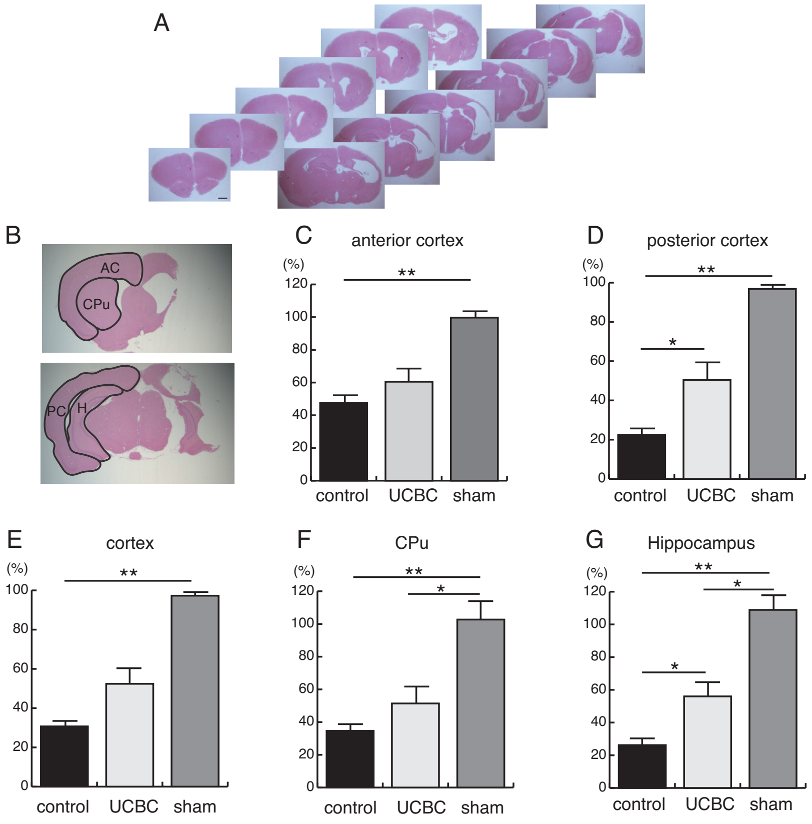

Supplementary Figure S1: Comparison of infarct volumes in various regions of the brain.

(A) Representative HE staining of every 100th paraffin-embedded sections.

(B) Using the HE-stained coronal sections, the estimated residual volumes of anterior cortex (AC, from Bregma+5mm to Bregma), posterior cortex (PC, from Bregma to Bregma-5mm), caudate putamen (CPu), and hippocampus (H) were compared. (C – G) Estimated residual volumes of AC (C), PC (D), cortex (E), CPu (F), and hippocampus (G). Each residual volume was designated as a proportion of ipsilateral to contralateral hemisphere volume and represented as a percentage. The estimated residual volume of each region was calculated with similar way to the estimated residual brain volume (Fig. 2B) described in supplementary information. The absolute value in each region was shown in Supplementary Table S1. The residual volume in the PC and hippocampus of SCE-UCBC-treated rats was significantly larger than that of control rats. Data are expressed as means ± SEM. *; $p < 0.05$, **; $p < 0.01$. (sham-operated, n = 5; control n = 15; UCBC, n = 14).

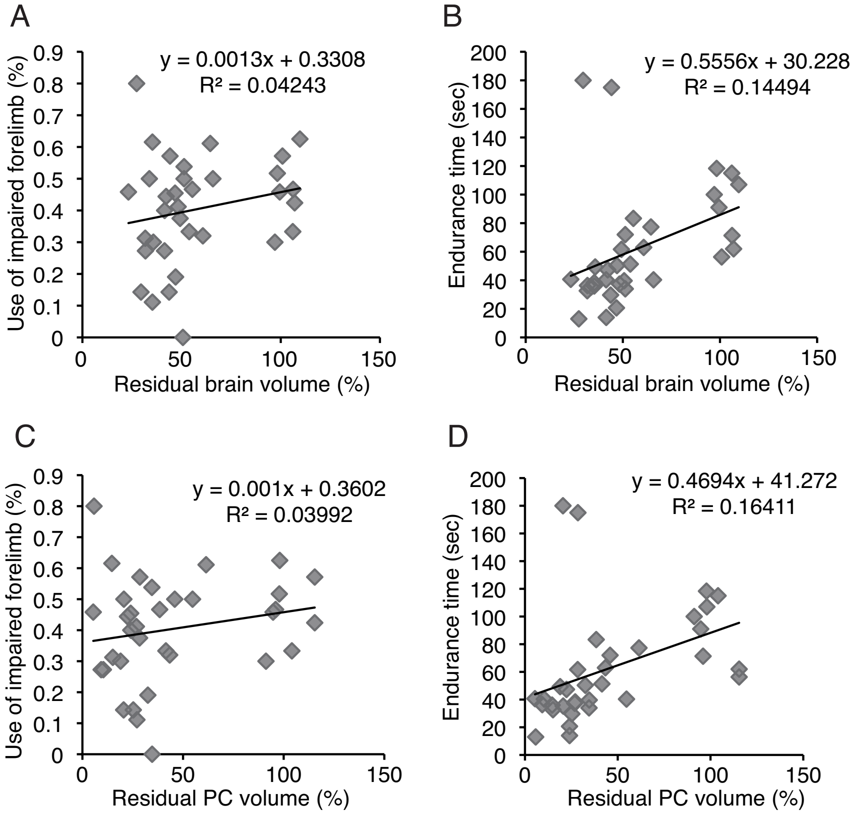

Supplementary Figure S2: Correlation between residual brain volume and motor function.

The correlation between the estimated residual brain volume (Fig. 2B) or posterior cortex (PC) volume (Suppl. Fig. S1D) evaluated by HE staining and motor function evaluated by the behavioral study (Fig. 2C and 2D) were analyzed in all rats of the three experimental groups. Scatter plot of estimated residual brain volume vs. the cylinder test (A), estimated residual brain volume vs. the rotarod test (B), estimated residual PC volume vs. the cylinder test (C), and estimated residual PC volume vs. the rotarod test (D). The result of linear regression analysis and its R-squared are shown in each panel. The endurance time on the rotarod had some correlation with the residual brain volume (B) or residual PC volume (D).

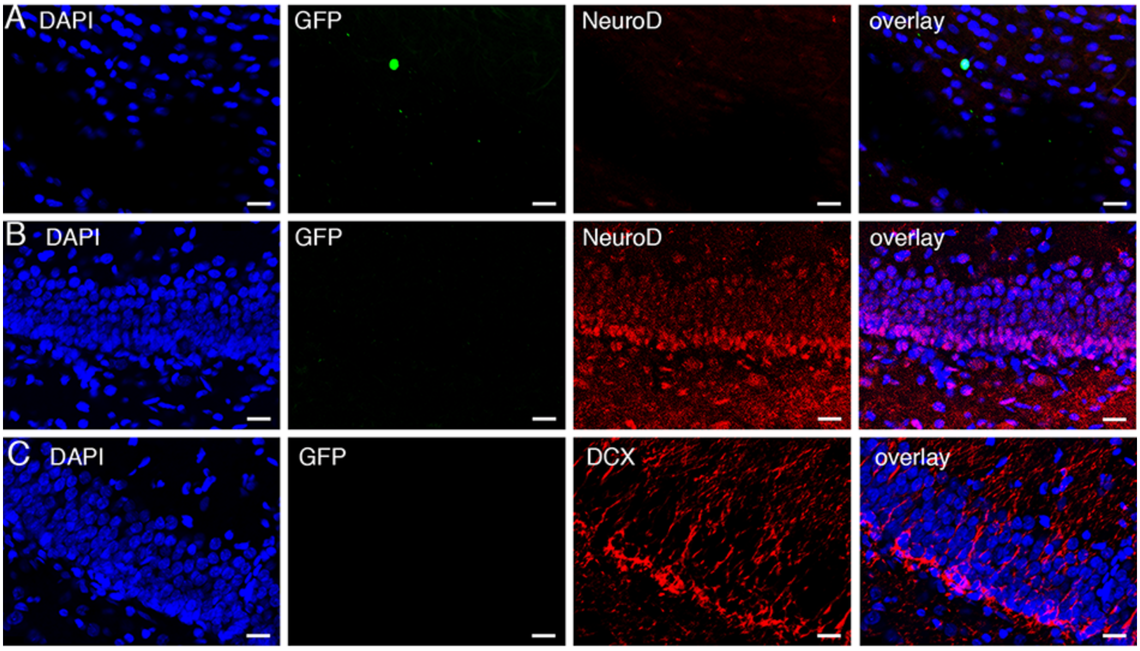

Supplementary Figure S3: The majority of intraperitoneally injected SCE-UCBCs in the brain of HI-treated rats were NeuroD- and DCX-negative.

Representative photomicrographs of the double staining of GFP/NeuroD (A and B) and GFP/DCX(C) in brains of HI-treated rats intraperitoneally injected with GFP-labeled SCE-UCBCs 7 days after injection. DAPI (blue), anti-GFP antibody (green), and anti-NeuroD (A and B) or DCX(C) antibody (red) staining, rightmost panel, overlay. None of GFP+ cells were positive for NeuroD (0 out of 10 GFP-positive cells) or DCX (0 out of 9 GFP-positive cells). Although NeuroD+ (B) or DCX+(C) cells were mainly observed in the DG, we failed to detect either GFP+/NeuroD+ or GFP+/DCX+ cells in the DG. Bar, A - C, 20 μm.

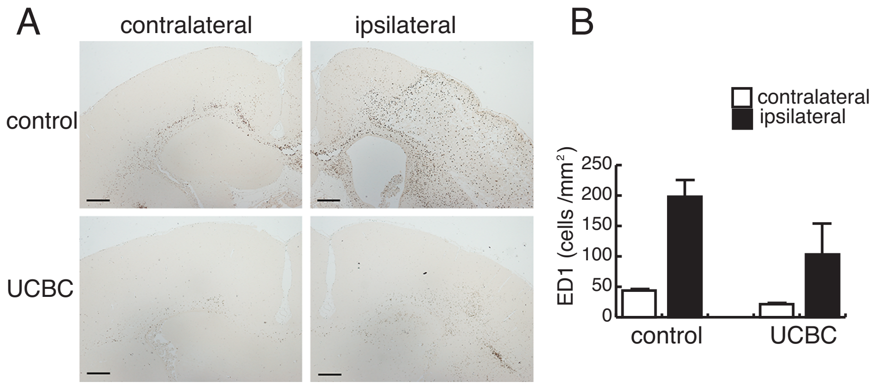

Supplementary Figure S4: Effect of injection of SCE-UCBCs on the immune response one week after insult.

(A) Representative photomicrographs of the peri-infarct area stained with anti-ED1 antibodies in the contralateral (left panels) and ipsilateral (right panels) hemispheres of the control rat (upper panels) and the SCE-UCBC-treated rat (lower panels) one week after insult. In contrast to the results at 3 weeks after insult (Fig. 7B), ED1-positive cells were present in both hemispheres of both groups at 1 week after insult. Bar, 100 μm. (B) The numbers of ED1-positive cells in the contralateral (open boxes) and ipsilateral hemisphere (closed boxes). The number of ED1-positive cells in both hemispheres of SCE-UCBC-treated rats decreased compared to that of control rats. Data are expressed as means ± SEM. (control n = 2; UCBC, n = 2).

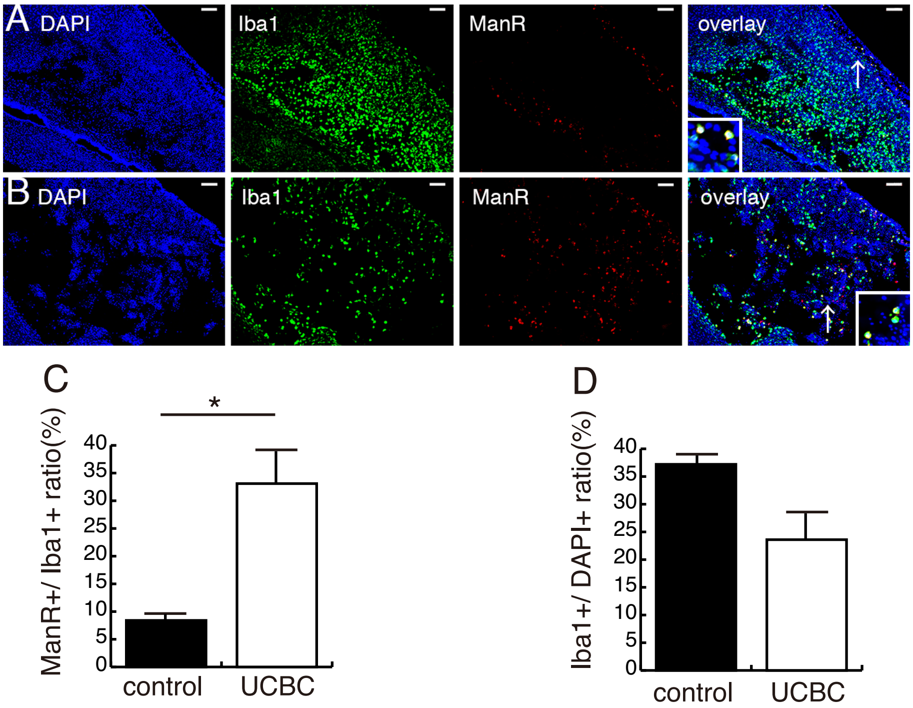

Supplementary Figure S5: Effect of injection of SCE-UCBCs on the M2 microglia/macrophage response 9 days after administration.

(A, B) Representative photomicrographs of the ipsilateral infarct area stained with anti-Iba1 (pan-microglia/macrophage marker, green) and anti-mannose receptor (M2 microglia/macrophage marker, red) antibodies in a control rat (A) and SCE-UCBC-treated rat (B) 9 days after administration. DAPI (blue), rightmost panel, overlay. Many mannose-receptor-positive cells have accumulated in the lesion area in the SCE-UCBC-treated rat. Insets show higher magnification views. Arrows indicate the area magnified. Bar, 100 μm. (C) The percentage of mannose receptor+/Iba1+ cells in the SCE-UCBC-treated rats (open boxes) was increased compared to control rats (closed boxes). (D) The percentage of Iba1+/DAPI+ cells was not significantly different. Data are expressed as means ± SEM. *; $p < 0.05$. (control n = 3; UCBC, n = 4).

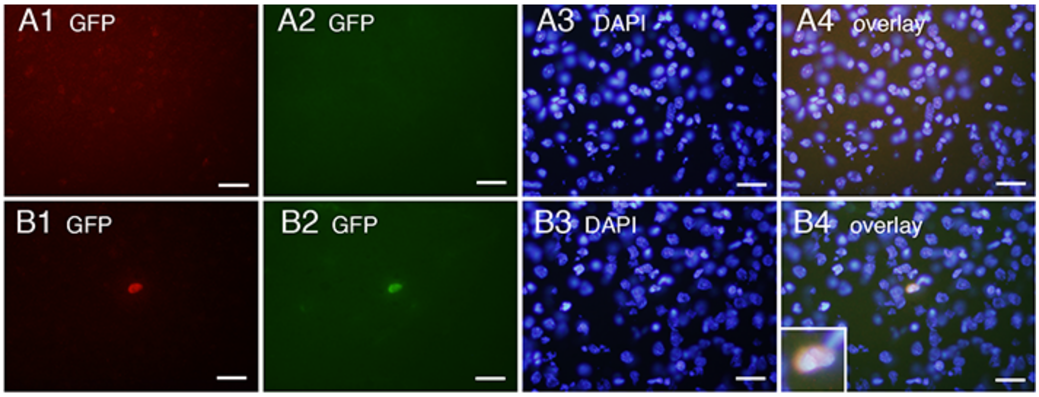

Supplementary Figure S6: Specificity of GFP antibodies.

Double staining of two kinds of GFP antibodies in a frozen section of the brain of a PBS-injected control rat (A) and an SCE-UCBC-treated rat (B) 7 days after insult. A1and B1, rabbit polyclonal anti-GFP antibodies (red), A2 and B2, mouse monoclonal anti-GFP antibodies (green), A3 and B3, DAPI (blue), A4 and B4, overlay.

(A) A GFP signal was not detected in the brain of the control rat. The GFP-positive cell in B is the same as that shown in Fig. 3D. These cells were photographed using a fluorescence microscope (Nikon, Eclipse E800). Bar, 50 μm.
